# Dimethylammonium Cation-Induced 1D/3D Heterostructure for Efficient and Stable Perovskite Solar Cells

**DOI:** 10.3390/molecules27217566

**Published:** 2022-11-04

**Authors:** Xianfang Zhou, Chuangye Ge, Xiao Liang, Fei Wang, Dawei Duan, Haoran Lin, Quanyao Zhu, Hanlin Hu

**Affiliations:** 1 State Key Laboratory of Advanced Technology for Materials Synthesis and Processing, School of Materials Science and Engineering, Wuhan University of Technology, Wuhan 430070, China; 2 Hoffmann Institute of Advanced Materials, Postdoctoral Innovation Practice Base, Shenzhen Polytechnic, Nanshan District, Shenzhen 518055, China

**Keywords:** perovskite solar cells, low-dimensional perovskite, two-step method

## Abstract

Mixed-dimensional perovskite engineering has been demonstrated as a simple and useful approach to achieving highly efficient and more-durable perovskite solar cells (PSCs), which have attracted increasing research interests worldwide. In this work, 1D/3D mixed-dimensional perovskite has been successfully obtained by introducing DMAI via a two-step deposition method. The additive DMA^+^ can facilitate the crystalline growth and form 1D DMAPbI_3_ at grain boundaries of 3D perovskite, leading to improved morphology, longer charge carrier lifetime, and remarkably reduced bulk trap density for perovskite films. Meanwhile, the presence of low-dimension perovskite is able to prevent the intrusion of moisture, resulting in enhanced long-term stability. As a result, the PSCs incorporated with 1D DMAPbI_3_ exhibited a first-class power conversion efficiency (PCE) of 21.43% and maintained 85% of their initial efficiency after storage under ambient conditions with ~45% RH for 1000 h.

## 1. Introduction

Halide perovskite solar cells (PSCs) have attracted significant attention due to their impressive potential for next-generation photovoltaic technology. They possess the merits of economical cost, capacity for large-scale fabrication, and remarkable power conversion efficiency (PCE) [1,2,3,4,5,6,7,8,9,10,11,12]. Since the first PSC fabricated by Kojima et al. in 2009 [1], numerous achievements have been accomplished to raise the PCE from 3.8% to 25.8% [5]. Despite inspiring development in past decades, the application of PSCs is severely restricted because of their poor stability against moisture, heat, and UV light, attributed to the inherent structural characteristics of conventional (three dimensional) perovskites [13,14,15,16]. To be specific, inevitable defects which form during fabrication process of PSCs and are located at grain boundaries and interfaces can not only serve as the center of trap-assisted non-radiative recombination, but also induce the decomposition of perovskite films in humid conditions and consequently decrease the efficiency and long-term stability of PSCs [17,18,19,20,21,22]. On the other hand, their severe toxicity is a potential threat to the environment and humankind [23].

To address these issues, various strategies have been attempted, such as additive engineering [23,24,25,26,27,28], interfacial modification [29,30,31,32,33,34], solvent engineering [2,35] and so on. Among them, the incorporation of one-dimensional (1D) perovskite has drawn great attention due to its unique characteristics in terms of stability [36,37,38,39]. The typical structural configuration of 1D perovskite can be described as the adjacent [PbX_6_]^4−^ octahedra surrounded by A-site cations that are corner-sharing, edge-sharing, or face-sharing to form the “shoulder to shoulder” arrangement. The hydrophobic large-size organic spacer cations at the A-site can effectively prevent the destructive effect of moisture on the perovskite lattice [37,40]. On the other hand, this natural structure leads to intrinsic shortcomings such as high binding energy, poor charge conductivity, and low film quality [41,42,43]. Therefore, the combination of 1D and 3D perovskites has been extensively studied by introducing large-size organic cations into the precursor solution of 3D perovskite via a one-step or two-step method to form a mixed heterojunction-based perovskite. Post-treatment deposition of a thin layer of organic cations on either the top or bottom of the 3D perovskite film constructs a layered multidimensional 1D/3D perovskite film. Both cases are environmentally stable features with promising photovoltaic performance. Bi et al. modified methylammonium lead iodide (MAPbI_3_) employing 1,1,1-trifluoro-ethyl ammonium iodide (FEAI) to obtain enhanced efficiency and stability of PSCs [36]. Fan et al. constructed flexible 1D/3D hybrid perovskite structures employing 2-(1H-pyrazol-1-yl) pyridine (PZPY) in formamidinium lead iodide (FAPbI_3_) with surprising thermodynamic self-healing ability, leading to long-term stability [44]. In addition, Yu et al. incorporated hydrazinium (HA) into (FAPbI_3_) to obtain stable α-(FAPbI_3_) through the formation of a 1D/3D hybrid dimensional structure [45]. Liu et al. used PbI_2_-bipydine (BPy) to achieve ideal lattice matching and alleviate ion migration [46]. In the work of Bi et al., 2-diethylaminoethylchloride hydrochloride (DEAECCl) was applied to form 1D perovskite, which played an important template role in facilitating the crystalline growth of 1D/3D perovskite structures [47]. Li et al. demonstrated the incorporation of benzimidazole cations (Bn^+^) can not only effectively induce the crystallization process of 1D/3D perovskite with preferred orientation but also suppress unbalanced charge carrier extraction, thus resulting in a stable lattice with significantly inhibited ion migration and ultra-long-term stability [40]. Hu’s group constructed a 1D/3D mixed-dimensional perovskite hetero-structure by introducing trimethylsulfonium iodide (Me_3_SI) to lead halide 3D perovskites; the target films exhibited improved morphology and crystallinity and substantially reduced carrier recombination, consequently enhancing the performance of corresponding devices [48]. Most recently, Zhao et al. adopted 1-ethyl-3-methylimidazolium trifluoroacetate (EMIMTFA) ionic liquid into MAPbI_3_ to achieve multi-level passivation through formed stable 1D EMIMPbI_3_ perovskites distributed at the upper and buried interfaces, and bulk phase 1D/3D perovskite, which significantly passivated bulk and interface defects and promoted carrier transfer, which enhanced PCE and long-term stability [49]. Even though numerous valuable achievements have been obtained, 1D perovskites still suffer from intrinsic poor and anisotropic charge conductivity, hence further exploration for a new cation that possesses the potential to form 1D perovskite and can improve photovoltaic performance and stability simultaneously is pretty essential.

Recently, a large organic cation—dimethylammonium (DMA)—has been comprehensively investigated, especially in all-inorganic PSCs. Kanatzidis et al. revealed that the acidic hydrolysis of N, N-dimethylformamide (DMF) induced by hydroiodic acid (HI) leads to the formation of DMA, which eventually stabilizes the CsPbI_3_ black perovskite phase [50]. Snaith et al. reported that DMA can act as an A-site cation to incorporate with CsPbI_3_, creating a hybrid perovskite of Cs_x_DMA_1-x_PbI_3_, which is more stable in ambient conditions than pure CsPbI_3_ [51]. Pang’s group systematically investigated the chemical composition and phase evolution of dimethylamine iodide (DMAI)-induced CsPbI_3_ perovskite through a precisely controlled thermal annealing process. Furthermore, they employed an optimized strategy to successfully construct a CsPbI_3_/DMA_0.15_Cs_0.85_PbI_3_ bulk heterojunction structure, which facilitated the charge separation and collection process and reduced carrier recombination loss simultaneously, and a remarkably high PCE of over 20% was achieved [52,53]. Ning et al. introduced DMAI to MAPbI_3_ as a secondary amine to increase the rigidity of the perovskite structure and realize a more capable lattice match, resulting in the effective suppression of defects at interfaces; the corresponding inverted devices exhibited an exceptional PCE of 21.6% [54]. Liu et al. introduced DMA to Cs-stabilized FAPbI_3_ perovskite to fabricate printable PSCs; the additional DMA regulated the Fermi level and produced a suitable band structure, enabling the PCE to reach 17.47% [55]. The abovementioned publications have demonstrated that DMA can effectively enhance the film quality of perovskite, leading to an improved photovoltaic performance in various aspects. However, the formation of low-dimension/3D perovskite has rarely been investigated, even though DMAPbI_3_ has been proven to be 1D perovskite structure [56,57,58].

In this work, we have introduced DMAI into the precursor solution to prepare mixed-dimensional perovskite and systematically investigate its specific effect on photovoltaic performance and long-term stability. All results revealed that DMAI could facilitate the crystallization process and coordinate with excessive PbI_2_ to form 1D DMAPbI_3_, which has a face-sharing connection of [PbI_6_]^4−^ octahedra. Moreover, the formation of 1D perovskite tends to locate at grain boundaries, leading to a reduced bulk trap density, efficient passivation of defects, and promotion of charge carrier transfer capability. Moreover, the presence of 1D perovskite significantly retarded the unfavorable intrusion of moisture, hence leading to the improved long-term stability of mixed-dimensional perovskites. As a result, the PSCs modified with 1D DMAPbI_3_ achieved a first-rate efficiency of 21.43%, whereas the unencapsulated device retained 85% of its initial efficiency after aging under ambient condition with ~45% RH for 1000 h.

## 2. Results and Discussion

**Figure 1:**
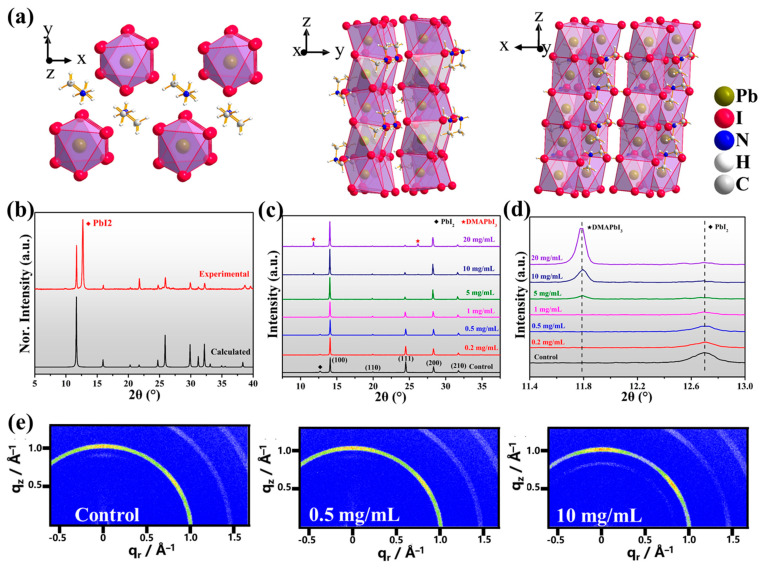
(**a**–**c**) Crystal structure of 1D DMAPbI_3_ perovskite along different directions. (**b**) Comparison of the calculated and experimental XRD patterns of DMAPbI_3_. (**c**,**d**) XRD patterns of perovskite films treated with different concentrations of DMAI. (**e**) GIWAXS images of three typical samples: control, 0.5 mg/mL representing low concentration, and 10 mg/mL representing high concentration.

We prepared pure 1D perovskite and mixed-dimensional perovskite films via a two-step method, where PbI_2_ and organic salt precursor solutions were sequentially deposited on the substrates by spin-coating and annealed to form target perovskite films. Various concentrations of DMAI were dissolved in IPA with the typical organic ammonium halides. More experimental details are stated in the experimental section. The crystal structure and crystallographic parameters of 1D perovskite with the chemical formula of DMAPbI_3_ (CCDC: 1497287) are shown in Figure 1a and Table S1, which consists of 1D chains of face-sharing [PbI_6_]^4−^ octahedra with large DMA^+^ cations [59]. X-ray diffraction (XRD) patterns were carried out to confirm the formation of DMAPbI_3_ and reveal the effect of DMAI on the crystallization process of perovskite.

As shown in Figure 1b, the XRD pattern of the deposited 1D DMAPbI_3_ perovskite film matches exactly with the calculated pattern from its single-crystal data; the strong peak located at 12.7° can be assigned as the excess PbI_2_. Figure 1c shows the XRD patterns of mixed-dimensional perovskite films: five characteristic diffraction peaks at 14.1°, 20.0°, 24.4°, 28.2°, and 31.8° are clearly observed, corresponding to the (100), (110), (111), (200), and (210) planes of 3D perovskite films [55]. For DMAI-treated perovskite films, no significant changes were reflected in the XRD patterns after adding small amounts of DMAI; as the concentration increased, another new diffraction peak appeared and gradually increased at 11.8°, which was attributed to 1D DMAPbI_3_ perovskites [57,58]. Meanwhile, the remnant PbI_2_ peaks were almost eliminated when the concentration of DMAI was more than 5 mg/mL, suggesting that additional DMAI in the precursor solution can react with excess PbI_2_ to form 1D/3D perovskite. Additionally, the maximum diffraction peaks attributed to (100) planes of 3D perovskite gradually shifted to a lower degree after addition of DMAI, as shown in Figure S1, indicating lattice expansion caused by substituting large cations into the perovskite A-site [60]. We carried out 2D grazing-incidence wide-angle X-ray scattering (GIWAXS) measurements to investigate the crystal structure and orientation of mixed-dimensional perovskite films. Figure 1e and Figure S2 show the GIWAXS images and integrated intensity of corresponding samples; the 1D/3D samples exhibit a barely changed intensity of diffraction rings at q_z_ = 1 Å^−1^, which corresponds to the (100) lattice plane of 3D perovskite. In contrast, remarkably diminished intensity of PbI_2_ diffraction spots at q_z_ = 0.9 Å^−1^ were observed. In addition, a new diffraction ring at q_z_ = 0.85 Å^−1^ that can be assigned as 1D DMAPbI_3_ perovskite was observed in high-concentration samples. In the following discussion, the additional concentration of DMAI in modified samples was 0.5 mg/mL, unless otherwise noted.

**Figure 2:**
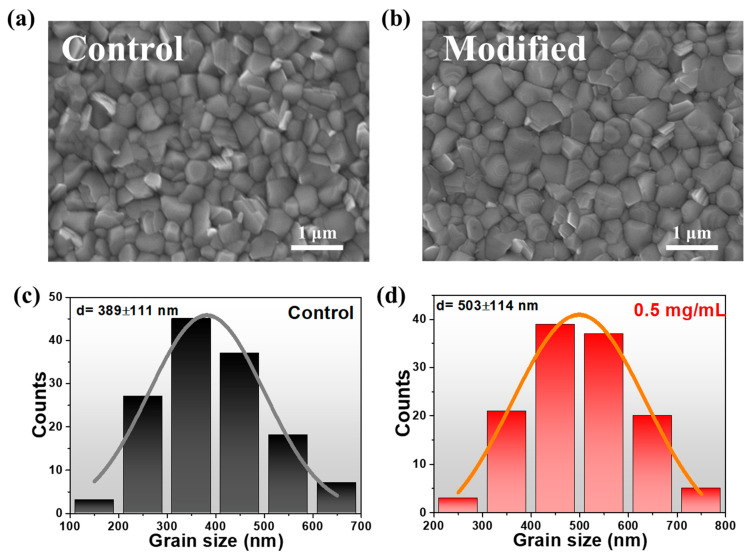
Top-view SEM images of control (**a**) and 1D/3D modified (**b**) perovskite films and corresponding statistical diagrams of crystallite size for control (**c**) and 1D/3D modified (**d**) perovskite film.

The existence of 1D DMAPbI_3_ in perovskite films and its influence on surface morphology are also characterized by top-view scanning electron microscopy (SEM) images, as shown in Figure 2.

It is obvious that numerous lead iodide grains were distributed in the control sample, whereas fewer similar grains were observed in the 1D/3D modified samples as the content of DMAI continuously increased, and finally disappeared. As the concentration further increased, several fiber-shaped perovskite crystals formed (as shown in Figure S3), and the residual PbI_2_ completely converted into 1D nanofiber perovskite crystals. When the addition was 10 mg/mL, we found more rod-shaped crystals form at the grain boundaries in morphology images. Therefore, it can be deduced that 1D perovskite was most likely located at the grain boundaries (GBs) for the samples with at low concentration addition of DMAI, which was beneficial to reducing non-radiative recombination through suppression and passivation of defects at GBs. To study the effect of DMAI on the crystallite size of perovskite, we measured the crystallite size, as seen in Figure 2a,b; the statistical results are illustrated in Figure 2c,d. The size ranged from 100 nm to 700 nm for the control sample while it increased to between 200 to 800 nm with the addition of DMAI. After fitting by the equation:

$$y=y_{0}+\frac{A}{W\sqrt{\pi /2}}e^{-2\frac{{\left(x-x_{c}\right)}^{2}}{w^{2}}}$$

we obtained the average crystallite size of 389 ± 111 nm and 503 ± 114 nm for the control and modified samples, respectively. These results indicate that DMAI could facilitate the crystallization process with the reduction of GBs.

**Figure 3:**
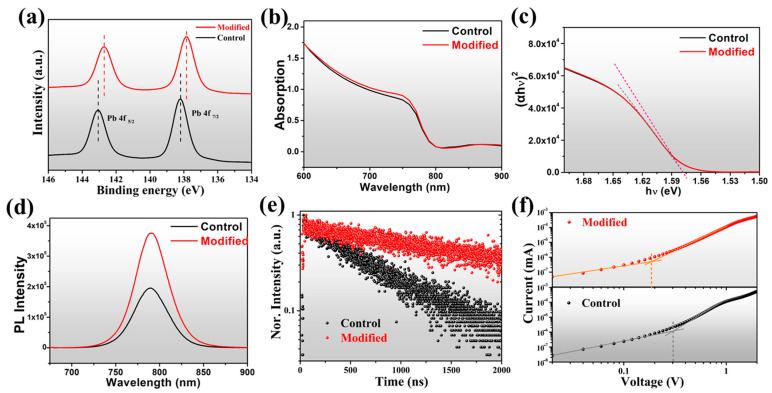
(**a**) XPS of Pb 4f core-level spectra for control and modified films. (**b**) UV–Vis absorption spectra and (**c**) Tauc-Plot spectra of perovskite films. (**d**) SSPL and (**e**) TRPL spectra of perovskite films. (**f**) Dark J-V curves of electron-only devices.

X-ray photoelectron spectroscopy (XPS) was used to investigate the effect of DMAI on the surface chemistry properties of perovskite films. As shown in Figure 3a, two dominant peaks of Pb^2+^ 4f_7/2_ and Pb^2+^ 4f_5/2_ are located at 138.3 eV and 143.1 eV in the control film; both peaks shifted about 0.4 eV toward the lower-energy region when modified with DMAI, which was ascribed to interaction between uncoordinated Pb^2+^ and DMA^+^ cation ligands [32].

To further investigate the impact of 1D DMAPbI_3_ on optical and electronic properties of mixed-dimensional perovskite films, ultraviolet–visible (UV-Vis) absorption, steady-state photoluminescence (SSPL), and time-resolved photoluminescence (TRPL) measurements were conducted. Figure 3b shows the UV-Vis absorption spectra of the perovskite films. All samples exhibited an absorption threshold around 800 nm and no apparent shifts occurred. Meanwhile, the Tauc-Plot spectra in Figure 3c reveal that the optical bandgap of control is estimated to be about 1.57 ± 0.02 eV, which is almost the same as the modified sample with incorporation of DMAI, suggesting that the addition of a small amount of DMAI has negligible effect on the optical bandgap of mixed 1D/3D perovskite, which has been proven in previous research [47]. Figure 3d shows that the addition of DMAI increases the SSPL intensity, which was attributed to the improved charge transport and suppressed non-radiative recombination in 1D/3D perovskite films with reduced trap density. Similarly, the bandgap calculated from PL was around 1.51 ± 0.05 eV for perovskite with and without the addition of DMAI (Figure S5b), which was smaller than that of 1.57 ± 0.03 eV calculated from the UV-Vis absorption result. The decrease in the bandgap could be attributed to the existence of a Stokes shift in the perovskite materials. The charge carrier lifetime of control and modified films was characterized by TRPL spectra (Figure 3e); the excitation source wavelength was 440 nm, and the decay curves were fitted with an exponential function. The modified film showed a much longer lifetime (1993.7 ns) over the control film (657.4 ns). The enhanced PL intensity and improved carrier lifetime imply the efficient passivation of defects in the mixed-dimensional perovskite films, which can improve device performance.

We adopted the space charge limited current (SCLC) method to systematically quantify the trap density of perovskite films. The dark J-V characteristic curves of electron-only devices with a configuration of glass/ITO/SnO_2_/perovskite/PCBM/Au are shown in Figure 3f. The trap-state density can be calculated by the equation: Nt = 2*ε*_0_*ε*_r_ *V*_TFL_/(*qL*^2^), where *ε*_0_, *ε_r_*, and *V*_TFL_ are the vacuum permittivity, the relative dielectric constant (*ε*_r_ = 46.9), and the trap-filled limited voltage, respectively; *q* is the elementary charge, and *L* is the film thickness. The *V*_TFL_ of modified film (0.18 V) was lower than that of the control sample (0.30 V), which corresponds to the reduced trap density from 4.04 × 10^15^ cm^−3^ to 2.42 × 10^15^ cm^−3^, indicating that the incorporation of DMAI effectively reduced electron trap density and diminished trap-assisted non-radiative recombination [47].

**Figure 4:**
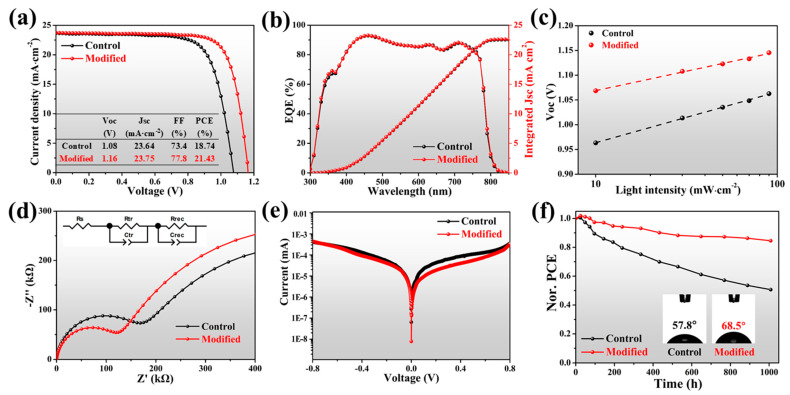
(**a**) Forward-scanning J-V curves of the control and modified devices under simulated AM 1.5 G illumination of 100 mW∙cm^−2^. (**b**) EQE spectra and (**c**) light-intensity-dependent *V_oc_* curves of PSCs. (**d**) Nyquist plots and (**e**) dark current leakage curves of PSCs. (**f**) long-term stability of normalized PCE of unencapsulated PSCs aged at ambient conditions; insets: water contact angles for control and modified.

To evaluate the photovoltaic (PV) performance of DMAI-modified PSCs, devices with a typical planar structure of glass/ITO/SnO_2_/perovskite/Spiro-OMeTAD/Au were fabricated and characterized. The optimum concentration of DMAI was 0.5 mg/mL; the current density voltage (J-V) curves of the control and modified devices are shown in Figure 4a and Figure S7, along with corresponding PV parameters listed in the insets and Table S2.

The best 1D/3D modified device exhibited a remarkably improved open-circuit voltage (*V*_oc_) of 1.16 V, compared with that of 1.08 V for the control device. Meanwhile, the short-circuit current density (*J*_sc_) and fill factor (FF) were enhanced from 23.64 mA∙cm^−2^ and 73.4% to 23.75 mA∙cm^−2^ and 77.8%, respectively, resulting in a significant improvement of PCE from 18.74% to 21.43%. Furthermore, the 1D/3D device showed less hysteresis (2.1%) than the 5.4% of the control device. Figure 4b shows the external quantum efficiency (EQE) spectra and the integrated *J*_sc_ of corresponding devices; the modified device exhibited a maximum EQE value of 93.1% within the wavelength range of 300 to 900 nm. Significantly, the incorporation of 1D perovskite enhanced the EQE value at 300–380 nm, similar phenomena to which have been reported in previous work [40,49,61]. The integrated *J*_sc_ calculated from EQE spectra reached 22.55 mA∙cm^−2^ and 22.64 mA∙cm^−2^ for control and modified devices, respectively. To study the reproducibility of our devices, we followed the method mentioned in the previous report [62]; the statistical distribution of the PV parameters from 15 individual devices with different concentrations of DMAI are summarized in Figure S8, revealing that the introduction of 1D perovskite in PSCs improves reproducibility, and the increment of PCE is owed primarily to the enhanced *V*_oc_ and FF.

To further investigate the mechanism of these improvements caused by the incorporation of 1D perovskite, we adopted light intensity-dependent *Voc* measurement, electrochemical impedance spectroscopy (EIS), and dark current measurement. As shown in Figure 4c, the modified PSC shows a smaller slope of 1.32 *k_B_T*/*q* compared to 1.72 *k_B_T*/*q* for the control device. Herein, the deviation between the calculated slope and unity *k_B_T*/*q* (where *k_B_* is the Boltzmann constant, *T* is the temperature, and *q* is the elementary charge) reflects the rate of trap-assisted Shockley –Read–Hall recombination; a smaller slope indicates a less trap-assisted non-radiative recombination with the addition of 1D perovskite [63,64,65]. Two semicircles can be found in each EIS curve; as shown in Figure 4d, each EIS curve can be classified to successively reflect charge transfer resistance (*R*_tr_) and recombination resistance (*R*_rec_) [66]. It is obvious that 1D/3D perovskite devices exhibited decreased *R*_tr_ and increased *R*_rec_, indicating the promoted charge transfer and inhibited charge recombination, thus leading to improved *V*_oc_ and *J*_sc_. Figure 4e shows the J-V curves in dark conditions, the lower leakage current of modified PSC compared to control sample indicating the notable suppressed charge recombination and promoted charge extraction attributed to the prominent reduction of trap density and efficient passivation of bulk defects, which simultaneously leads to the increase in FF [67,68]. All the results of the modified PSCs are in good agreement with the excellent properties of the corresponding films as stated above, indicating the incorporation of 1D perovskite significantly improves the PV performance of PSCs.

In addition, enhanced long-term stability of PSCs is expected to be achieved for the 1D/3D mixed perovskite device. The hydrophobicity of pristine and modified perovskite films is certified by water contact angle measurement, as shown in the insets in Figure 4f. A larger water contact angle was displayed for modified film than for the control film, indicating the existence of 1D perovskite, which is beneficial against moisture and thus inhibited destruction of the lattice. We have tracked the PV performance of unencapsulated devices under ambient conditions with ~45% RH for 1000 h (Figure 4f). The modified device exhibited a prolonged long-term stability while maintaining 85% of its original PCE. However, the efficiency of the control sample dramatically decreased to about 50% of its initial value after aging for1000 h.

## 3. Conclusions

In summary, we have successfully synthesized 1D/3D mixed-dimensional perovskite via a two-step method by simply introducing DMAI to the precursor solution of organic cation halide. The addition of DMAI regulated crystalline growth and reduced residual PbI_2_ simultaneously. Meanwhile, the incorporated 1D perovskite, which tended to locate at the grain boundaries, could significantly suppress the formation of bulk defects and retard the invasion of moisture. Consequently, the optimized film properties of inhibited non-radiative recombination, improved charge carrier transfer, and enhanced long-term stability has been achieved. The corresponding devices exhibited a first-class PCE of 21.43% and maintained 85% of initial efficiency after aging under ambient conditions with ~45% RH for 1000 h.

## Data Availability

Not applicable.

## Supplementary Materials

The following supporting information can be downloaded at: https://www.mdpi.com/article/10.3390/molecules27217566/s1, Figure S1: XRD patterns zoomed in (100) planes of perovskite films treated with different concentration of DMAI; Figure S2: The radially integrated intensity from GIWAXS data of corresponding perovskite film samples; Figure S3: Top-view SEM images of perovskite films with 5 mg/mL, 10 mg/mL and 20 mg/mL DMAI; Figure S4: UV-vis absorption spectra (a) and corresponding Tauc-Plots (b) of perovskite films with different concentration of DMAI; Figure S5: (a) SSPL spectra of perovskite films with different concentration of DMAI. (b) The tauc plot calculated from the PL results with and without 0.5 mg/mL addition of DMAI; Figure S6: TRPL spectra of perovskite films with different concentration of DMAI; Figure S7: Forward and reverse scanning J-V curves of the champion control and modified devices; Figure S8: Statistical distribution of photovoltaic parameters for PSCs incorporated with different concentration of DMAI. Table S1: Crystallographic parameters for DMAPbI_3_; Table S2: Photovoltaic parameters of the champion control and modified PSCs under forward and reverse scans.

## Sample Availability

Samples of the compounds are available from the authors.

## References

[1] Kojima A., Teshima K., Shirai Y., Miyasaka T. (2009). Organometal halide perovskites as visible-light sensitizers for photovoltaic cells. J. Am. Chem. Soc..

[2] Bu T., Li J., Li H., Tian C., Su J., Tong G., Ono L.K., Wang C., Lin Z., Chai N. (2021). Lead halide-templated crystallization of methylamine-free perovskite for efficient photovoltaic modules. Science.

[3] Li Z., Li B., Wu X., Sheppard S.A., Zhang S., Gao D., Long N.J., Zhu Z. (2022). Organometallic-functionalized interfaces for highly efficient inverted perovskite solar cells. Science.

[4] Jeong J., Kim M., Seo J., Lu H., Ahlawat P., Mishra A., Yang Y., Hope M.A., Eickemeyer F.T., Kim M. (2021). Pseudo-halide anion engineering for α-FAPbI3 perovskite solar cells. Nature.

[5] Min H., Lee D.Y., Kim J., Kim G., Lee K.S., Kim J., Paik M.J., Kim Y.K., Kim K.S., Kim M.G. (2021). Perovskite solar cells with atomically coherent interlayers on SnO_2_ electrodes. Nature.

[6] Yoo J.J., Seo G., Chua M.R., Park T.G., Lu Y., Rotermund F., Kim Y.-K., Moon C.S., Jeon N.J., Correa-Baena J.-P. (2021). Efficient perovskite solar cells via improved carrier management. Nature.

[7] Xiong Z., Chen X., Zhang B., Odunmbaku G.O., Ou Z., Guo B., Yang K., Kan Z., Lu S., Chen S. (2022). Simultaneous Interfacial Modification and Crystallization Control by Biguanide Hydrochloride for Stable Perovskite Solar Cells with PCE of 24.4%. Adv. Mater..

[8] Hu H., Qin M., Fong P.W.K., Ren Z., Wan X., Singh M., Su C., Jeng U., Li L., Zhu J. (2021). Perovskite Quantum Wells Formation Mechanism for Stable Efficient Perovskite Photovoltaics—A Real-Time Phase-Transition Study. Adv. Mater..

[9] Wang F., Wai-Keung Fong P., Ren Z., Xia H.-L., Zhou K., Wang K., Zhu J., Huang X., Liu X.-Y., Wang H. (2022). In-depth understanding of ionic liquid assisted perovskite film formation mechanism for two-step perovskite photovoltaics. J. Energy Chem..

[10] Zhou B., Liu Z., Fang S., Zhong H., Tian B., Wang Y., Li H., Hu H., Shi Y. (2021). Efficient White Photoluminescence from Self-Trapped Excitons in Sb3+/Bi3+-Codoped Cs2NaInCl6Double Perovskites with Tunable Dual-Emission. ACS Energy Lett..

[11] Wang F., Duan D., Singh M., Sutter-Fella C.M., Lin H., Li L., Naumov P., Hu H. (2022). Ionic Liquid Engineering in Perovskite Photovoltaics. Energy Environ. Mater..

[12] Cho N., Li F., Turedi B., Sinatra L., Sarmah S.P., Parida M.R., Saidaminov M.I., Murali B., Burlakov V.M., Goriely A. (2016). Pure crystal orientation and anisotropic charge transport in large-area hybrid perovskite films. Nat. Commun..

[13] Domanski K., Correa-Baena J.P., Mine N., Nazeeruddin M.K., Abate A., Saliba M., Tress W., Hagfeldt A., Grätzel M. (2016). Not All That Glitters Is Gold: Metal-Migration-Induced Degradation in Perovskite Solar Cells. ACS Nano.

[14] Domanski K., Roose B., Matsui T., Saliba M., Turren-Cruz S.H., Correa-Baena J.P., Carmona C.R., Richardson G., Foster J.M., De Angelis F. (2017). Migration of cations induces reversible performance losses over day/night cycling in perovskite solar cells. Energy Environ. Sci..

[15] Domanski K., Alharbi E.A., Hagfeldt A., Grätzel M., Tress W. (2018). Systematic investigation of the impact of operation conditions on the degradation behaviour of perovskite solar cells. Nat. Energy.

[16] Khenkin M.V., Katz E.A., Abate A., Bardizza G., Berry J.J., Brabec C., Brunetti F., Bulović V., Burlingame Q., Di Carlo A. (2020). Consensus statement for stability assessment and reporting for perovskite photovoltaics based on ISOS procedures. Nat. Energy.

[17] Wang Q., Chen B., Liu Y., Deng Y., Bai Y., Dong Q., Huang J. (2017). Scaling behavior of moisture-induced grain degradation in polycrystalline hybrid perovskite thin films. Energy Environ. Sci..

[18] Heo S., Seo G., Lee Y., Seol M., Kim S.H., Yun D.-J., Kim Y., Kim K., Lee J., Lee J. (2019). Origins of High Performance and Degradation in the Mixed Perovskite Solar Cells. Adv. Mater..

[19] Chen Q., Deng K., Shen Y., Li L. (2022). Stable one dimensional (1D)/three dimensional (3D) perovskite solar cell with an efficiency exceeding 23%. InfoMat.

[20] Wang D., Wright M., Elumalai N.K., Uddin A. (2016). Stability of perovskite solar cells. Sol. Energy Mater. Sol. Cells.

[21] Wang F., Ge C., Duan D., Lin H., Li L., Naumov P., Hu H. (2022). Recent Progress in Ionic Liquids for Stability Engineering of Perovskite Solar Cells. Small Struct..

[22] Ren J., Liu T., He B., Wu G., Gu H., Wang B., Li J., Mao Y., Chen S., Xing G. (2022). Passivating Defects at the Bottom Interface of Perovskite by Ethylammonium to Improve the Performance of Perovskite Solar Cells. Small.

[23] Fradi K., Bouich A., Slimi B., Chtourou R. (2022). Towards improving the optoelectronics properties of MAPbI_3_(_1−x_)B_3x_/ZnO heterojunction by bromine doping. Optik.

[24] Li L., Xu X., Xiao L., Jiang W., Zhao J., Kong X., Zou G. (2021). Symmetrical Conjugated Molecular Additive for Defect Passivation and Charge Transfer Bridge in Perovskite Solar Cells. ACS Appl. Energy Mater..

[25] Wang Y., Yang Y., Li N., Hu M., Raga S.R., Jiang Y., Wang C., Zhang X.L., Lira-Cantu M., Huang F. (2022). Ionic Liquid Stabilized Perovskite Solar Modules with Power Conversion Efficiency Exceeding 20%. Adv. Funct. Mater..

[26] Zhang F., Zhu K. (2020). Additive Engineering for Efficient and Stable Perovskite Solar Cells. Adv. Energy Mater..

[27] Liu S., Guan Y., Sheng Y., Hu Y., Rong Y., Mei A., Han H. (2020). A Review on Additives for Halide Perovskite Solar Cells. Adv. Energy Mater..

[28] Liang X., Zhou X., Ge C., Lin H., Satapathi S., Zhu Q., Hu H. (2022). Advance and prospect of metal-organic frameworks for perovskite photovoltaic devices. Org. Electron..

[29] Han T., Tan S., Xue J., Meng L., Lee J., Yang Y. (2019). Interface and Defect Engineering for Metal Halide Perovskite Optoelectronic Devices. Adv. Mater..

[30] Wang Y., Zhang Z., Tao M., Lan Y., Li M., Tian Y., Song Y. (2020). Interfacial modification towards highly efficient and stable perovskite solar cells. Nanoscale.

[31] Li Y., Xie H., Lim E.L., Hagfeldt A., Bi D. (2022). Recent Progress of Critical Interface Engineering for Highly Efficient and Stable Perovskite Solar Cells. Adv. Energy Mater..

[32] Li M.H., Sun T.G., Shao J.Y., Wang Y.D., Hu J.S., Zhong Y.W. (2021). A sulfur-rich small molecule as a bifunctional interfacial layer for stable perovskite solar cells with efficiencies exceeding 22%. Nano Energy.

[33] Chen J., Yang Y., Dong H., Li J., Zhu X., Xu J., Pan F., Yuan F., Dai J., Jiao B. (2022). Highly efficient and stable perovskite solar cells enabled by low-dimensional perovskitoids. Sci. Adv..

[34] Liu G., Zheng H., Ye J., Xu S., Zhang L., Xu H., Liang Z., Chen X., Pan X. (2021). Mixed-Phase Low-Dimensional Perovskite-Assisted Interfacial Lead Directional Management for Stable Perovskite Solar Cells with Efficiency over 24%. ACS Energy Lett..

[35] Bu T., Ono L.K., Li J., Su J., Tong G., Zhang W., Liu Y., Zhang J., Chang J., Kazaoui S. (2022). Modulating crystal growth of formamidinium–caesium perovskites for over 200 cm2 photovoltaic sub-modules. Nat. Energy.

[36] Bi D., Gao P., Scopelliti R., Oveisi E., Luo J., Grätzel M., Hagfeldt A., Nazeeruddin M.K. (2016). High-performance perovskite solar cells with enhanced environmental stability based on amphiphile-modified CH3NH3PbI3. Adv. Mater..

[37] Lin H., Zhou C., Tian Y., Siegrist T., Ma B. (2018). Low-Dimensional Organometal Halide Perovskites. ACS Energy Lett..

[38] Fu Y., Zhu H., Chen J., Hautzinger M.P., Zhu X.Y., Jin S. (2019). Metal halide perovskite nanostructures for optoelectronic applications and the study of physical properties. Nat. Rev. Mater..

[39] Rahaman M.Z., Ge S., Lin C.-H., Cui Y., Wu T. (2021). One-Dimensional Molecular Metal Halide Materials: Structures, Properties, and Applications. Small Struct..

[40] Zhan Y., Yang F., Chen W., Chen H., Shen Y., Li Y., Li Y. (2021). Elastic Lattice and Excess Charge Carrier Manipulation in 1D–3D Perovskite Solar Cells for Exceptionally Long-Term Operational Stability. Adv. Mater..

[41] Tsai H., Nie W., Blancon J.-C., Stoumpos C.C., Asadpour R., Harutyunyan B., Neukirch A.J., Verduzco R., Crochet J.J., Tretiak S. (2016). High-efficiency two-dimensional Ruddlesden–Popper perovskite solar cells. Nature.

[42] Koh T.M., Shanmugam V., Schlipf J., Oesinghaus L., Müller-Buschbaum P., Ramakrishnan N., Swamy V., Mathews N., Boix P.P., Mhaisalkar S.G. (2016). Nanostructuring Mixed-Dimensional Perovskites: A Route Toward Tunable, Efficient Photovoltaics. Adv. Mater..

[43] Yang R., Li R., Cao Y., Wei Y., Miao Y., Tan W.L., Jiao X., Chen H., Zhang L., Chen Q. (2018). Oriented Quasi-2D Perovskites for High Performance Optoelectronic Devices. Adv. Mater..

[44] Fan J., Ma Y., Zhang C., Liu C., Li W., Schropp R.E.I., Mai Y. (2018). Thermodynamically Self-Healing 1D-3D Hybrid Perovskite Solar Cells. Adv. Energy Mater..

[45] Yu S., Liu H., Wang S., Zhu H., Dong X., Li X. (2021). Hydrazinium cation mixed FAPbI3-based perovskite with 1D/3D hybrid dimension structure for efficient and stable solar cells. Chem. Eng. J..

[46] Liu P., Xian Y., Yuan W., Long Y., Liu K., Rahman N.U., Li W., Fan J. (2020). Lattice-Matching Structurally-STable 1D@3D Perovskites toward Highly Efficient and Stable Solar Cells. Adv. Energy Mater..

[47] Kong T., Xie H., Zhang Y., Song J., Li Y., Lim E.L., Hagfeldt A., Bi D. (2021). Perovskitoid-Templated Formation of a 1D@3D Perovskite Structure toward Highly Efficient and Stable Perovskite Solar Cells. Adv. Energy Mater..

[48] Ge C., Lu J.F., Singh M., Ng A., Yu W., Lin H., Satapathi S., Hu H. (2022). Mixed Dimensional Perovskites Heterostructure for Highly Efficient and Stable Perovskite Solar Cells. Sol. RRL.

[49] Wei N., Chen Y., Wang X., Miao Y., Qin Z., Liu X., Wei H., Zhao Y. (2022). Multi-Level Passivation of MAPbI 3 Perovskite for Efficient and Stable Photovoltaics. Adv. Funct. Mater..

[50] Ke W., Spanopoulos I., Stoumpos C.C., Kanatzidis M.G. (2018). Myths and reality of HPbI3 in halide perovskite solar cells. Nat. Commun..

[51] Marshall A.R., Sansom H.C., McCarthy M.M., Warby J.H., Ashton O.J., Wenger B., Snaith H.J. (2021). Dimethylammonium: An A-Site Cation for Modifying CsPbI3. Sol. RRL.

[52] Meng H., Shao Z., Wang L., Li Z., Liu R., Fan Y., Cui G., Pang S. (2020). Chemical Composition and Phase Evolution in DMAI-Derived Inorganic Perovskite Solar Cells. ACS Energy Lett..

[53] Sun X., Shao Z., Li Z., Liu D., Gao C., Chen C., Zhang B., Hao L., Zhao Q., Li Y. (2022). Highly efficient CsPbI3/Cs1-xDMAxPbI3 bulk heterojunction perovskite solar cell. Joule.

[54] Chen H., Wei Q., Saidaminov M.I., Wang F., Johnston A., Hou Y., Peng Z., Xu K., Zhou W., Liu Z. (2019). Efficient and Stable Inverted Perovskite Solar Cells Incorporating Secondary Amines. Adv. Mater..

[55] Zhao J., Wang H., Duan L., Lv T., Xiao B., Zhang J., Liu J., Zhang Y., Liu Q. (2022). Mechanism of the Dimethylammonium Cation in Hybrid Perovskites for Enhanced Performance and Stability of Printable Perovskite Solar Cells. Sol. RRL.

[56] Mancini A., Quadrelli P., Amoroso G., Milanese C., Boiocchi M., Sironi A., Patrini M., Guizzetti G., Malavasi L. (2016). Synthesis, structural and optical characterization of APbX3 (A=methylammonium, dimethylammonium, trimethylammonium; X=I, Br, Cl) hybrid organic-inorganic materials. J. Solid State Chem..

[57] Pei Y., Liu Y., Li F., Bai S., Jian X., Liu M. (2019). Unveiling Property of Hydrolysis-Derived DMAPbI3 for Perovskite Devices: Composition Engineering, Defect Mitigation, and Stability Optimization. iScience.

[58] Bian H., Wang H., Li Z., Zhou F., Xu Y., Zhang H., Wang Q., Ding L., Liu S., Jin Z. (2020). Unveiling the Effects of Hydrolysis-Derived DMAI/DMAPbI x Intermediate Compound on the Performance of CsPbI 3 Solar Cells. Adv. Sci..

[59] García-Fernández A., Bermúdez-García J.M., Castro-García S., Llamas-Saiz A.L., Artiaga R., López-Beceiro J., Hu S., Ren W., Stroppa A., Sánchez-Andújar M. (2017). Phase Transition, Dielectric Properties, and Ionic Transport in the [(CH_3_)_2_NH_2_]PbI_3_ Organic-Inorganic Hybrid with 2H-Hexagonal Perovskite Structure. Inorg. Chem..

[60] Eperon G.E., Stone K.H., Mundt L.E., Schloemer T.H., Habisreutinger S.N., Dunfield S.P., Schelhas L.T., Berry J.J., Moore D.T., Eperon G.E. (2020). The Role of Dimethylammonium in Bandgap Modulation for Stable Halide Perovskites. ACS Energy Lett..

[61] Yang N., Zhu C., Chen Y., Zai H., Wang C., Wang X., Wang H., Ma S., Gao Z., Wang X. (2020). An in situ cross-linked 1D/3D perovskite heterostructure improves the stability of hybrid perovskite solar cells for over 3000 h operation. Energy Environ. Sci..

[62] Bouich A., Marí-Guaita J., Soucase B.M., Palacios P. (2022). Manufacture of High-Efficiency and Stable Lead-Free Solar Cells through Antisolvent Quenching Engineering. Nanomaterials.

[63] Yang D., Zhou X., Yang R., Yang Z., Yu W., Wang X., Li C. (2016). Surface optimization to eliminate hysteresis for record efficiency planar perovskite solar cells. Energy Environ. Sci..

[64] Yang D., Yang R., Wang K., Wu C., Zhu X., Feng J., Ren X., Fang G., Priya S., Liu S.F. (2018). High efficiency planar-type perovskite solar cells with negligible hysteresis using EDTA-complexed SnO_2_. Nat. Commun..

[65] Chen J., Zhao X., Kim S., Park N. (2019). Multifunctional Chemical Linker Imidazoleacetic Acid Hydrochloride for 21% Efficient and Stable Planar Perovskite Solar Cells. Adv. Mater..

[66] Sonmezoglu S., Akin S. (2020). Suppression of the interface-dependent nonradiative recombination by using 2-methylbenzimidazole as interlayer for highly efficient and stable perovskite solar cells. Nano Energy.

[67] Chao L., Xia Y., Li B., Xing G., Chen Y., Huang W. (2019). Room-Temperature Molten Salt for Facile Fabrication of Efficient and Stable Perovskite Solar Cells in Ambient Air. Chem.

[68] Qiu J., Zheng Y., Xia Y., Chao L., Chen Y., Huang W. (2019). Rapid Crystallization for Efficient 2D Ruddlesden–Popper (2DRP) Perovskite Solar Cells. Adv. Funct. Mater..

