# Supplementary material for: Dimethylammonium Cation-Induced 1D/3D Heterostructure for Efficient and Stable Perovskite Solar Cells
Source: Molecules. 2022 Nov 4;27(21):7566. doi: 10.3390/molecules27217566 (PMC9656943; doi:10.3390/molecules27217566)
Supplement: Supplementary file 1 [file molecules-27-07566-s001.zip › molecules-1976964-supplementary.pdf]

molecules

Supporting Materials

Dimethylammonium Cation-Induced 1D/3D Heterostructure for Efficient and Stable Perovskite Solar Cells

Xianfang Zhou [1,2], Chuangye Ge [2], Xiao Liang [1,2], Fei Wang [1,2], Dawei Duan [2], Haoran Lin [2], Quanyao Zhu [1,*] and Hanlin Hu [2,*]

[1] State Key Laboratory of Advanced Technology for Materials Synthesis and Processing, School of Materials Science and Engineering, Wuhan University of Technology, Wuhan 430070, China
[2] Hoffmann Institute of Advanced Materials, Postdoctoral Innovation Practice Base, Shenzhen Polytechnic, Nanshan District, Shenzhen 518055, China

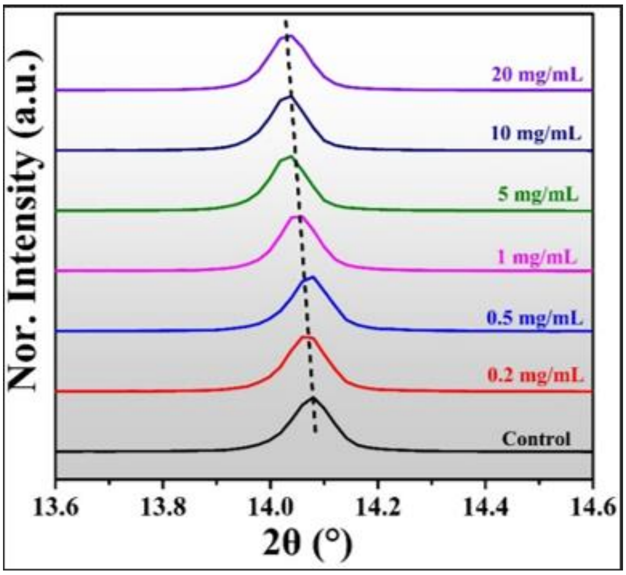

Figure S1. XRD patterns zoomed in (100) planes of perovskite films treated with different concentration of DMAI.

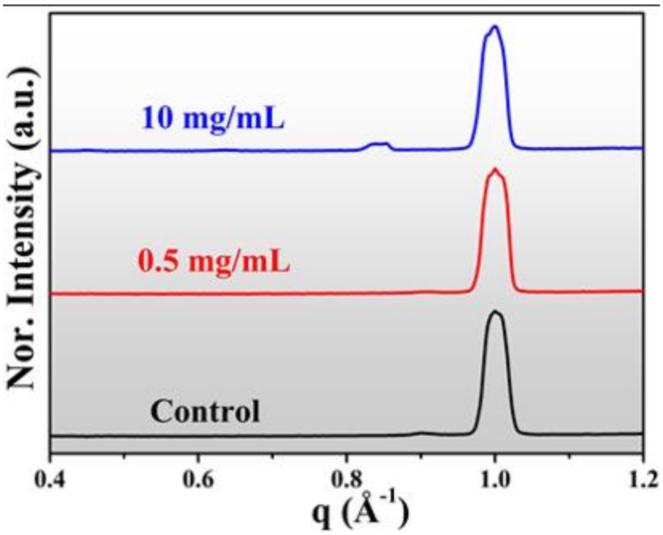

Figure S2. The radially integrated intensity from GIWAXS data of corresponding perovskite film samples.

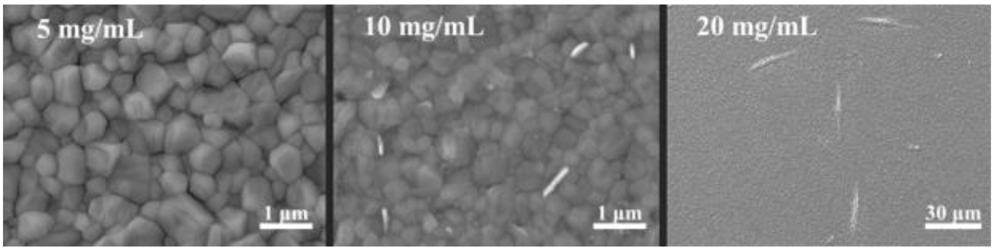

Figure S3. Top-view SEM images of perovskite films with 5 mg/mL, 10 mg/mL and 20 mg/mL DMAI. .

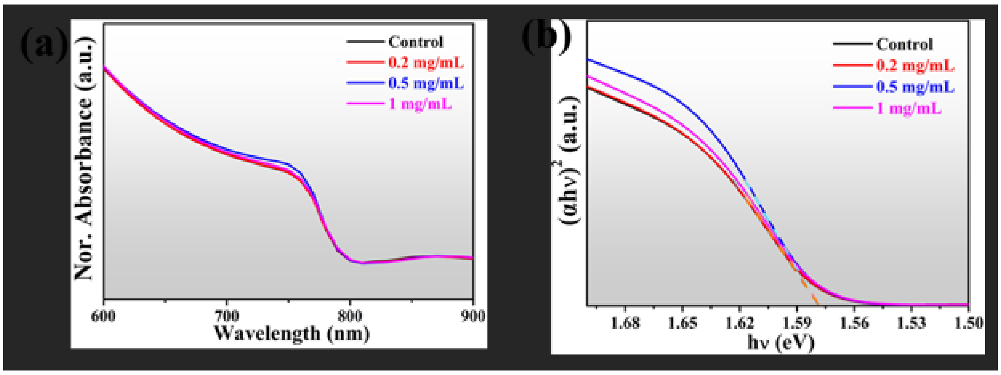

Figure S4. UV-vis absorption spectra (a) and corresponding Tauc-Plots (b) of perovskite films with different concentration of DMAI.

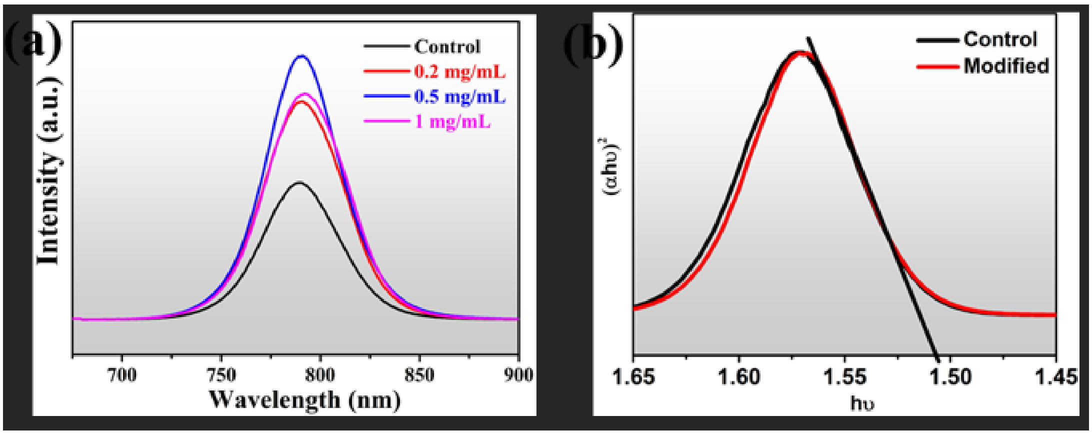

Figure S5. (a) SSPL spectra of perovskite films with different concentration of DMAI. (b) The tauc plot calculated from the PL results with and without 0.5 mg/mL addition of DMAI.

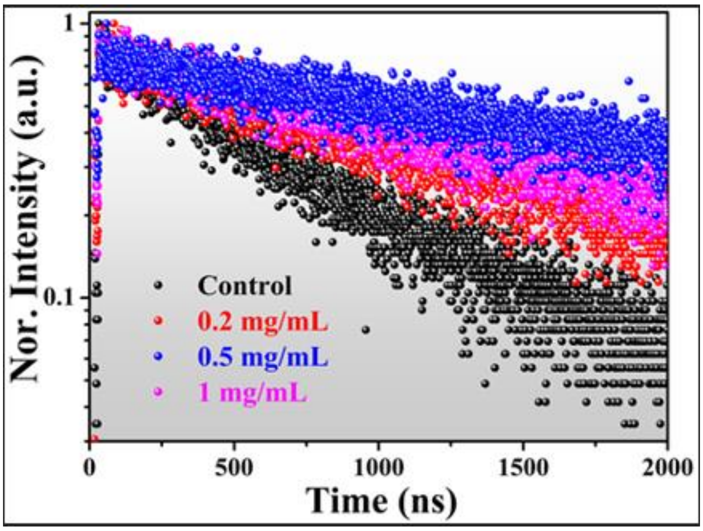

Figure S6. TRPL spectra of perovskite films with different concentration of DMAI.

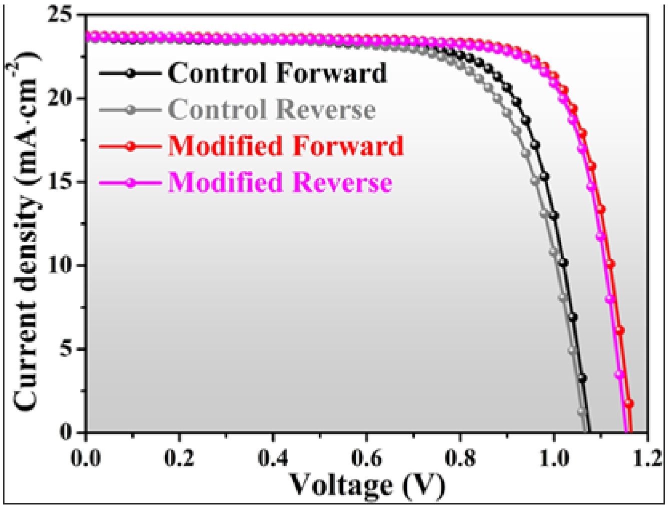

Figure S7. Forward and reverse scanning J-V curves of the champion control and modified devices.

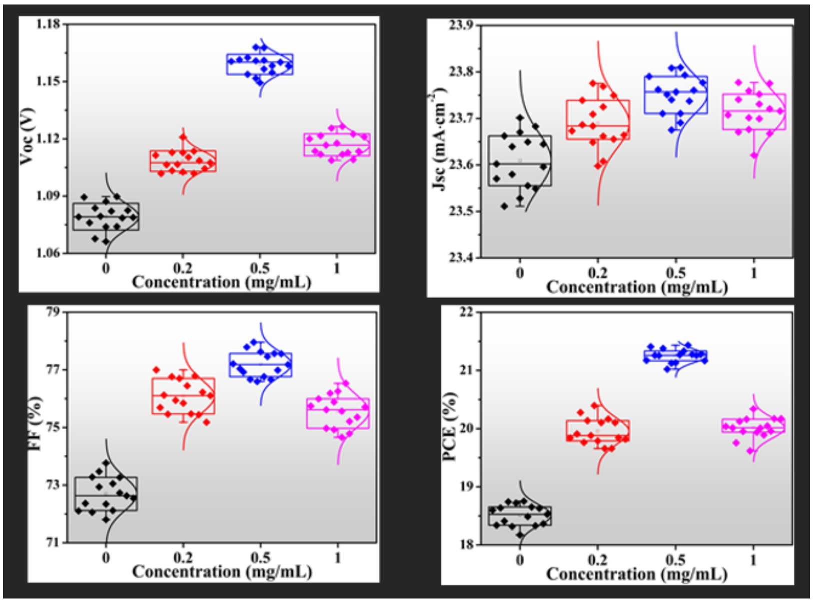

Figure S8. Statistical distribution of photovoltaic parameters for PSCs incorporated with different concentration of DMAI.

Table S1. Crystallographic parameters for $DMAPbI_3$.

**Compound**	**$DMAPbI_3$**
Empirical formula	$C_2H_8I_3NPb$
Formula weight/ g·mol^{-1}	634.01
Temperature (K)	100(2)
Crystal system	Hexagonal
Space group	$P2_1/c$
a/ Å	8.9303(6)
b/ Å	14.6910(9)
c/ Å	7.9790(5)
α/ °	90
β/ °	95.994(4)
γ/ °	90
Volume/ Å^3	1041.08(12)
CCDC numbers	1497287

Table S2. Photovoltaic parameters of the champion control and modified PSCs under forward and reverse scans.

	**Scan direction**	***Voc* (V)**	***Jsc* (mA·cm^{-2})**	**FF (%)**	**PCE (%)**
**Control**	**Forward**	**1.08**	**23.64**	**73.4**	**18.74**
	**Reverse**	**1.07**	**23.60**	**70.2**	**17.72**
**Modified**	**Forward**	**1.16**	**23.75**	**77.8**	**21.43**
	**Reverse**	**1.15**	**23.69**	**77.3**	**21.06**
